# Clinical Recommendations for Reducing the Risk of Cognitive Decline

**DOI:** 10.1093/geroni/igab046.707

**Published:** 2021-12-17

**Authors:** G Adriana Perez, Kelly O'Brien, Marwan Sabbagh, Michelle Bruno

**Affiliations:** 1 University of Pennsylvania School of Nursing, Phildelphia, Pennsylvania, United States; 2 UsAgainstAlzheimer's, Chicago, Illinois, United States; 3 Cleveland Clinic Nevada, Las Vegas, Nevada, United States; 4 Avalere Health, Washington, District of Columbia, United States

## Abstract

As much as 40% of dementia cases can be attributed to modifiable risk factors (Livingston et al., 2020). Much of that risk-reduction can be accomplished by changing behavior in midlife. In light of the emerging evidence that dementia may be preventable, UsAgainstAlzheimer’s convened a workgroup of national experts to develop new recommendations that primary care clinicians and general neurologists can use to initiate primary prevention conversations with their patients about cognitive decline. Few resources address steps that clinicians can take in their routine care to help patients reduce risk. Some relevant resources provide excellent guidance but tend to be more focused on early detection or slowing disease progression rather than primary prevention. The Risk Reduction Workgroup (RRWG) was convened to help address the need for clinicians to know how to discuss cognitive decline with their patients. The workgroup aligned on 11 recommendations for primary care clinicians and general neurologists. In addition the RRWG provide considerations for implementing the recommendations in clinical practice. The recommendations are mindful of social determinants of health, account for cultural differences, and are designed for general accessibility. This effort is part of a broader initiative by UsAgainstAlzheimer’s to address risk-reduction for cognitive decline and early interventions. Under the guidance of a multidisciplinary Provider Leadership Group consisting of representatives from some of the nation's largest health provider serving organizations, three independent workgroups are developing guidance and tools to assist providers in their clinical practice and improve health outcomes for patients at-risk for Alzheimer's and related dementias.

